# The JBEI quantitative metabolic modeling library (jQMM): a python library for modeling microbial metabolism

**DOI:** 10.1186/s12859-017-1615-y

**Published:** 2017-04-05

**Authors:** Garrett W. Birkel, Amit Ghosh, Vinay S. Kumar, Daniel Weaver, David Ando, Tyler W. H. Backman, Adam P. Arkin, Jay D. Keasling, Héctor García Martín

**Affiliations:** 1 Biological Systems and Engineering Division, Lawrence Berkeley National Laboratory, Berkeley, CA USA; 2 Joint BioEnergy Institute, Emeryville, CA USA; 3 Department of Chemical and Biomolecular Engineering, University of California, Berkeley, CA USA; 4 Department of Bioengineering, University of California, Berkeley, CA USA; 5 Environmental Genomics and Systems Biology Division, Lawrence Berkeley National Laboratory, Berkeley, CA USA; 6 School of Energy Science and Engineering, Indian Institute of Technology (IIT), Kharagpur, India; 7 Novo Nordisk Foundation Center for Biosustainability, Technical University of Denmark, Hørsholm, DK2970 Denmark; 8 DOE Agile BioFoundry, Emeryville, CA USA; 9 BCAM, Basque Center for Applied Mathematics, Bilbao, Spain

**Keywords:** Flux analysis, ^13^ C Metabolic Flux Analysis, -omics data, Predictive biology

## Abstract

**Background:**

Modeling of microbial metabolism is a topic of growing importance in biotechnology. Mathematical modeling helps provide a mechanistic understanding for the studied process, separating the main drivers from the circumstantial ones, bounding the outcomes of experiments and guiding engineering approaches. Among different modeling schemes, the quantification of intracellular metabolic fluxes (i.e. the rate of each reaction in cellular metabolism) is of particular interest for metabolic engineering because it describes how carbon and energy flow throughout the cell. In addition to flux analysis, new methods for the effective use of the ever more readily available and abundant -omics data (i.e. transcriptomics, proteomics and metabolomics) are urgently needed.

**Results:**

The jQMM library presented here provides an open-source, Python-based framework for modeling internal metabolic fluxes and leveraging other -omics data for the scientific study of cellular metabolism and bioengineering purposes. Firstly, it presents a complete toolbox for simultaneously performing two different types of flux analysis that are typically disjoint: Flux Balance Analysis and ^13^C Metabolic Flux Analysis. Moreover, it introduces the capability to use ^13^C labeling experimental data to constrain comprehensive genome-scale models through a technique called two-scale ^13^C Metabolic Flux Analysis (2S-^13^C MFA). In addition, the library includes a demonstration of a method that uses proteomics data to produce actionable insights to increase biofuel production. Finally, the use of the jQMM library is illustrated through the addition of several Jupyter notebook demonstration files that enhance reproducibility and provide the capability to be adapted to the user’s specific needs.

**Conclusions:**

jQMM will facilitate the design and metabolic engineering of organisms for biofuels and other chemicals, as well as investigations of cellular metabolism and leveraging -omics data. As an open source software project, we hope it will attract additions from the community and grow with the rapidly changing field of metabolic engineering.

**Electronic supplementary material:**

The online version of this article (doi:10.1186/s12859-017-1615-y) contains supplementary material, which is available to authorized users.

## Background

Metabolism is the set of physical and chemical processes through which living organisms extract energy and mass from their environment in order to sustain life [1]. Hence, an understanding of metabolism is fundamental in order to set the stage for, and understand the physical limitations of, every biological process. For example, metabolic disorders lie at the heart of a variety of human illnesses such as diabetes [2], obesity [3] and cancer [4].

Understanding of metabolism is also of central importance to the microbial production of drugs, bioproducts and biofuels through metabolic engineering [5]. This application of microbial bioengineering to produce biofuels and other commodity products has attracted significant attention due to its potential to reduce the CO_2_ emissions which cause climate change, reduce society’s reliance on fossil fuels for energy and chemicals, and provide for enhanced energy security. The production of commodity chemicals from lignocellulosic biomass is critical for achieving these goals, particularly in view of expected future population growth in emergent economies coupled with a marked improvement in their living standards [6].

Metabolic engineering rarely succeeds at the first attempt to introduce and activate a heterologous pathway in a host organism. Rather, most of the time in this process is spent troubleshooting the pathways for acceptable production [7]. Very often, the metabolic engineer diagnoses the problem in the bioengineered microbe by relying on a variety of -omics data (transcriptomics, proteomics, metabolomics) that are expected to provide an explanation of why the system does not behave as expected [8–10]. However, this diagnosing through -omics data is a non trivial process: often the amount of data is much larger than what metabolic engineers are typically trained to analyze, and it is non-trivial to extract actionable items (e.g. overexpress this specific gene or change that promoter) that will reliably improve the system’s behavior [11, 12]. To complicate matters, the reason for the underperformance of a given pathway may not rely on the pathway itself but on other parts of the host metabolism that affect the pathway’s performance in intricate and non-direct ways (e.g. cofactor availability or gene regulation) [13].

Mathematical modeling helps contextualize experimental data into a model that provides an explanation of the observed phenomena and provides predictions for changes in the system under study. Metabolic modeling, in particular, has been successfully used to e.g. increase product yield [14] or identify bottlenecks and competing pathways [15]. Moreover, data analysis and modeling is expected to increase in scope and importance as the creation of increasingly larger sets of -omics data sets are enabled by the rapid drop of DNA sequencing costs [16] and the availability of high-throughput mass spectrometry workflows [17, 18]. Hence, research institutes such as the Joint BioEnergy Institute (JBEI), tasked to produce the scientific and technological underpinnings that will enable lignocellulosic biofuels [19], have devoted research effort not only to develop drop-in biofuels [20] but also tools to speed up bioengineering through mathematical modeling.

Some of the mathematical tools created at JBEI to enable better prediction of bacterial and eukaryotic metabolism are presented here in the form of the JBEI Quantitative Metabolic Modeling library (jQMM). The jQMM library includes algorithms to measure and predict internal metabolic fluxes using three different techniques: ^13^C Metabolic Flux Analysis (^13^C MFA [21]), Flux Balance Analysis (FBA [22]) and two-scale ^13^ *C* Metabolic Flux Analysis (2S-^13^C MFA [23]). It also includes methods to produce actionable insights from -omics data to improve pathway yield [24], and methodologies for the flux analysis of microbial communities [25]. We hope to add to the jQMM library in the future tools and algorithms currently being developed at JBEI, as well other institutions who may consider contributing to this library.

## Methods

### Key capabilities

The JBEI QMM library includes code to perform the following techniques: FBA [22], ^13^C MFA [21], 2S-^13^C MFA [23], Principal Component Analysis of Proteomics (PCAP [24]), and ^13^C MFA for microbial communities [25]. Mathematical details for each method are provided in the supplementary material (Additional file 1). FBA, ^13^C MFA and 2S-^13^C MFA are techniques used to calculate internal metabolic fluxes; detailed explanations regarding the differences between each method can be found in Garcia Martin et al. [23]. This library contains sample demonstration scripts to, for example, replicate all the figures from Garcia Martin et al. in the form of Jupyter notebooks (see Table 1). The code in the jQMM library focuses on ^13^C MFA, 2S-^13^C MFA, since there is already an open source python-based library [26] for FBA. For ^13^C MFA there exists some closed-source packages (e.g. METRAN [27], INCA [28], 13CFLUX2 [29]), a few open-source packages (OPENFLUX [30] and OPENFLUX2 [31]) but no open source python-based libraries. For 2S-^13^C MFA, jQMM is the first public library.

**Table 1:** Table of iPython Notebooks

iPython Notebook number	Topic
A0	module tests
A1	Core demo
A2	Enhanced lists demo
A3	ReactionNetworks demo
A4	FluxModels demo
A5	GAMSclasses demo
A6	Predictions demo
A7	Labeling and DB demo
B1	FBA demo
B2	TCA ^13^ *C* MFA demo
B3	Toya data ^13^ *C* MFA demo
B4	Toya data 2S- ^13^ *C* MFA demo
B5	PCAP example
B6	^13^C MFA for microbial communities

### Implementation

**Fig. 1:**
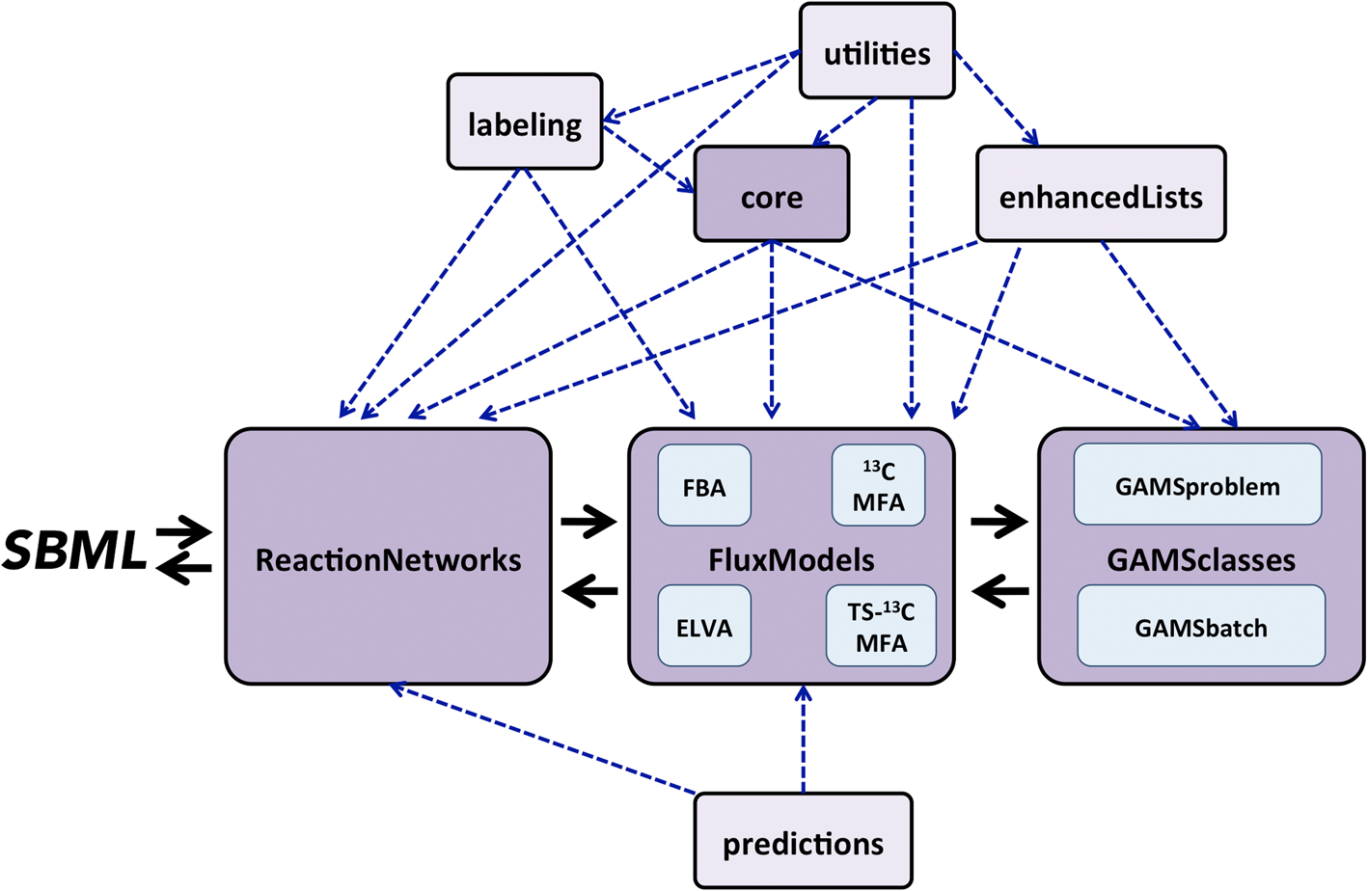
Diagram of jQMM library module relationships and typical flow for flux analysis. Flux calculations typically start with information stored in an SBML (Systems Biology Markup Language) file and translated into a reaction network. That reaction network is enclosed in a flux model of the appropriate type for the desired method (FBA, ^13^C MFA, 2S-^13^C MFA, ELVA). The flux model instance uses the GAMSclasses module in order to solve the appropriate optimization problem, and turns it back to the flux model instance, which stores the information as a reaction network or an SBML file. The core, labeling, utilities and enhancedLists modules are used by these “main workflow” modules (in *darker shade*). Predictions use information from reaction networks and flux models to make flux predictions for genetic modifications

**Fig. 2:**
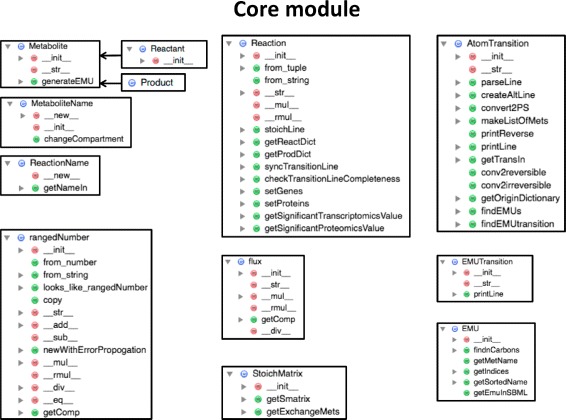
Core module class diagram. The core module contains the core classes for the rest of the library: classes for metabolite, reaction, flux, elementary metabolite units (EMU), atom transitions, emu transitions and stoichiometry matrices

**Fig. 3:**
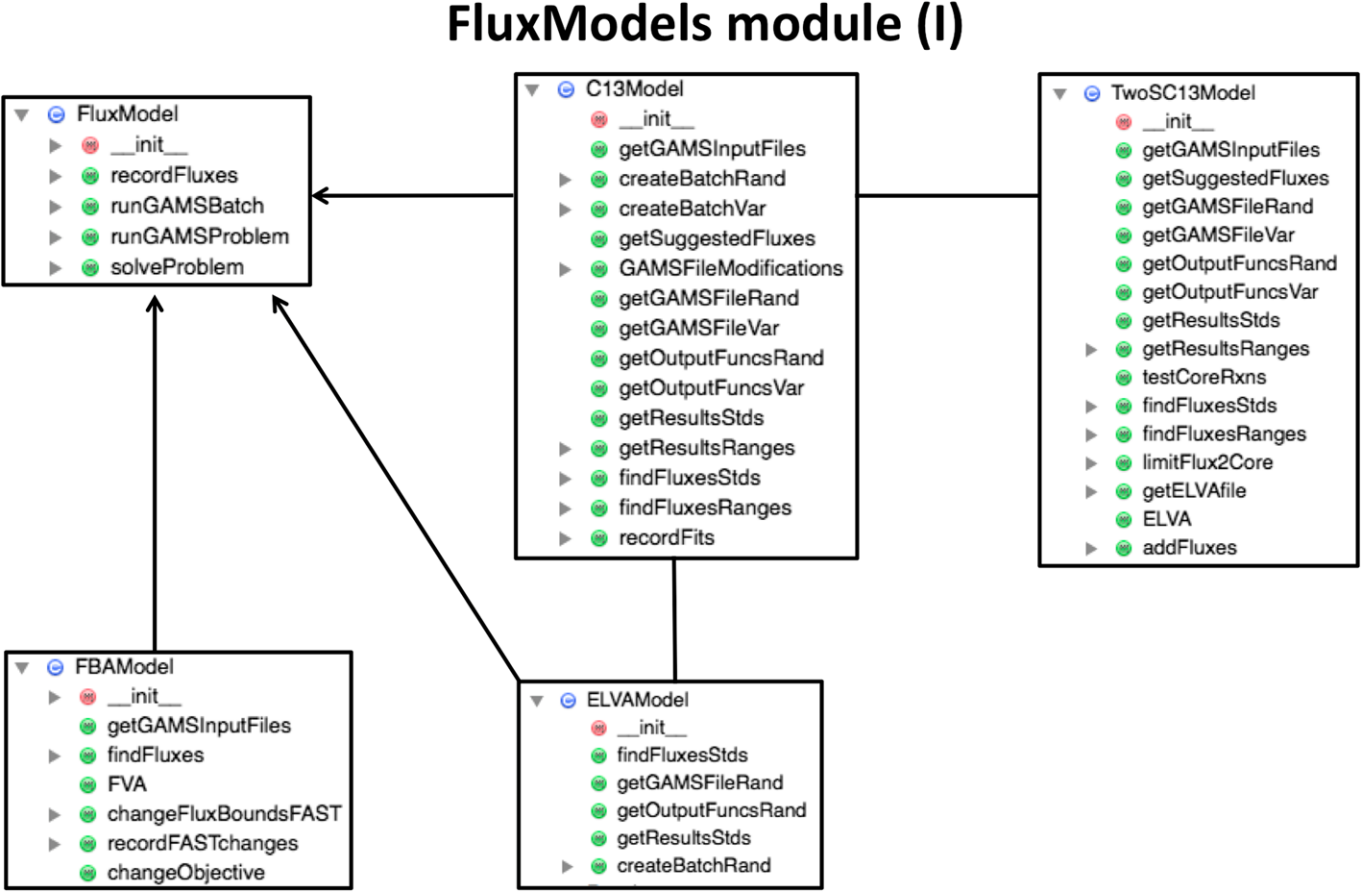
FluxModels module class diagram (part I). The FluxModels module contains classes for the different types of models used for each flux analysis type: FBA, ^13^C MFA, 2S-^13^C MFA, ELVA, etc. *Arrows* indicate derived classes

**Fig. 4:**
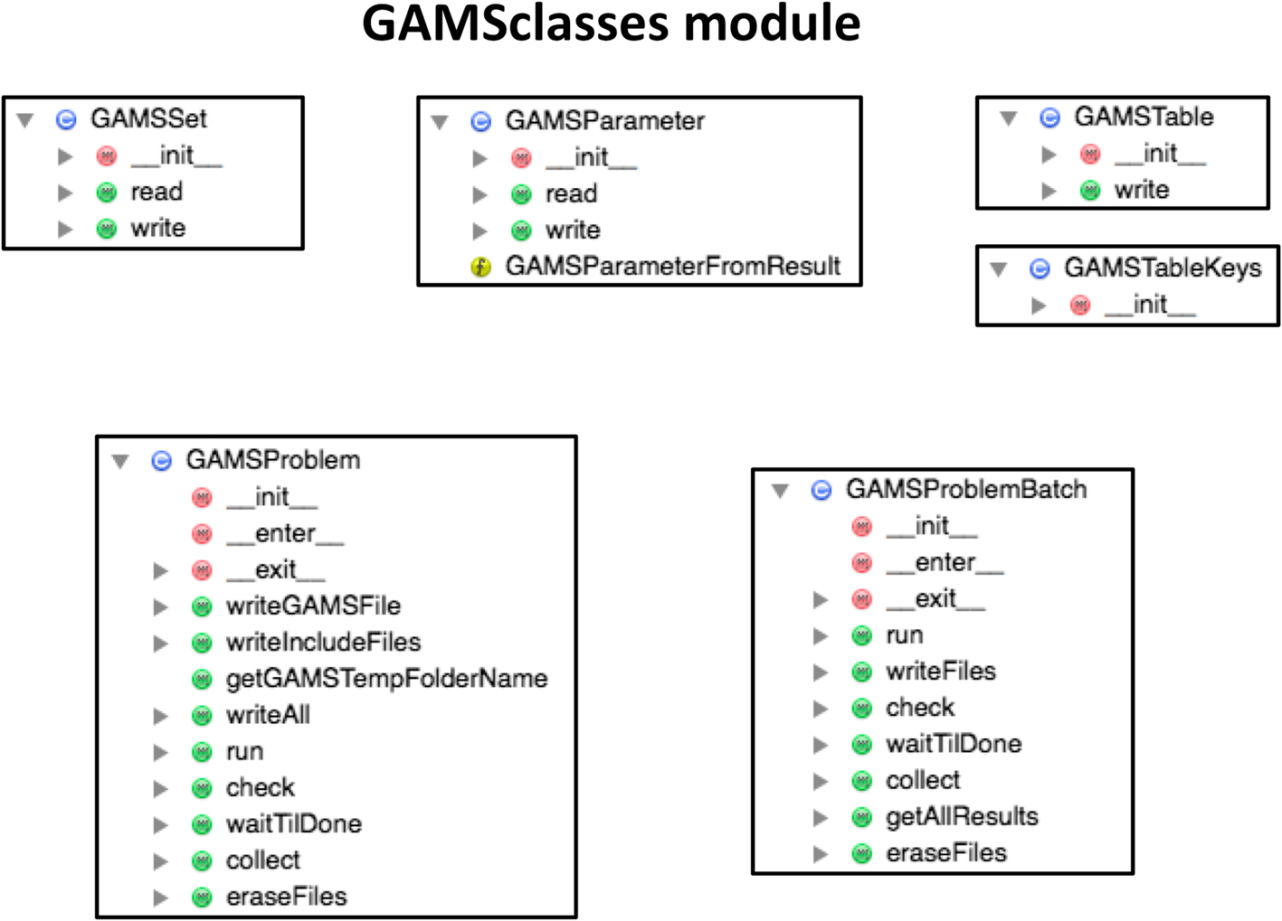
GAMSclasses module class diagram. The GAMSclasses module contains the classes needed to solve the optimization problems through the GAMS algebraic modeling system. Each of the optimization problems is described through a GAMS file, GAMS sets, GAMS parameters or GAMS tables, which are used as input for the GAMSproblem instance (see Fig. 1). GAMS batches are sets of GAMS problems, to be solved in *series* or *parallel*

jQMM is implemented in Python 2.7, and we have aimed to provide a code base that is as modular as possible, in order to facilitate understanding and reusability. The code is divided into a set of modules that incorporate classes and methods which will typically be used together. Figure 1 details the different modules and how they interact with each other for flux-based analysis. The following subsections provide a brief explanation of the purpose of each module and Figs. 2, 3 and 4 (and Additional file 1: Figures S1-S3) provide graphical depictions of each module together with their main classes and methods. Tutorials are provided for each module, along with unit tests.

In order to facilitate reproducibility, the lack of which represents one of the main problems afflicting biological research [32], all results and tutorials are provided in the form of Jupyter notebooks [33, 34] (see Table 1 and jupyter.org). This approach is meant to facilitate the reproduction of published results and the creation of new results based on these. The Jupyter notebooks are divided into two types: type A demonstrates the library’s capabilities and type B reproduces published results using code from the jQMM library (Table 1). To further enhance reproducibility we have provided a docker container in the github repository that includes all dependencies. Docker (http://www.docker.com) containers wrap a piece of software in a complete filesystem containing everything needed to run: code, systems libraries, systems tools and anything else that can be installed in a server. This guarantees that it will always run correctly and in the same way, regardless of the system environment it is running in. The jQMM docker container can be run on any computer and any cloud computing service such as Amazon Web Services (AWS), Google Cloud Platform or Microsoft Azure. The container does not include GAMS, CPLEX or CONOPT licences, which must be provided by the user.

### Modules

This section contains a succint explanation for each module and some of its classes and capabilities. A more detailed description of each class containing all possible functionality can be found in the Jupyter notebooks (Table 1) and the jQMM code on the github website (see availability below).

#### Core

The core module contains the classes that constitute the base for all other functionality (Fig. 2). These basic classes include the *Reaction* and *Metabolite* classes, along with the derived *Product* and *Reactant* classes. These classes are defined as required to describe labeling experiments and, as such, are different from equivalently named classes in COBRApy [26] (defined for FBA purposes). However, we expect in the future to derive these classes from the corresponding COBRApy classes. Other basic classes include the *flux* class, which is used to describe fluxes for a given reaction (See Jupyter notebook A1).

Core classes that describe concepts exclusively used in ^13^ *C* labeling experiments include the *atomTransition*, *emu* and *emuTransition* classes. The *atomTransition* class is used to hold carbon transition rules (information on the fate for each carbon atom in a reaction [23]), e.g.:



Note that this is different from the *Reaction* class, which would only hold information on the reaction name, products and reactants for a reaction:



By using two classes to differentiate transitions and reactions, we minimize confusion between two related but different concepts. The emu class describes elementary metabolite units (emus, [35]), which are used to efficiently calculate labeling patterns corresponding to a flux profile. Emus represent moieties comprising any distinct subset of a compound’s atoms: for example, if pyruvate consists of 3 carbons, an emu is a subset of any number of these 3 carbons: e.g. pyr_1_2, pyr_1_2_3 or pyr_2_3 are emus. The *EMUTransition* class describes how emus transition into each other for each reaction, for example for citrate synthase:



indicates that emus accoa_c_1_2 and oac_c_2 condense into emu cit_c_3_4_5. This initial decomposition of atom transitions into emus and emu transitions is fundamental to solving the forward problem (i.e. determine labeling from fluxes) in ^13^C MFA, and is provided as part of the core module, as shown in Jupyter notebook A2.

A final core class is the *rangedNumber* class, which describes floating point numbers with a lower and upper limit, reflecting confidence intervals. These type of numbers are used, for example, to hold information for a flux obtained from 2S- ^13^ *C* MFA, where we have a best fit value (0.5), plus upper (0.6) and lower limits (0.3) which represent the upper and lower flux values compatible with the experimental data (see Equation 23 in [23]):



#### ReactionNetworks

The ReactionNetworks module (see Additional file 1: Figure S1) contains the classes needed to store three types of reaction networks: stoichiometric (*reactionNetwork*, used for FBA), carbon transitions (*C13reactionNetwork*, for ^13^C MFA) and stochiometric with carbon transitions only for a defined core (*TSreactionNetwork*, for 2S-^13^C MFA). The first class contains the methods for manipulating purely stoichiometric reaction networks, such as those used in FBA, the second one contains the methods for dealing with carbon transition networks typically used in ^13^C MFA and the final class contains the methods used to manipulate networks where genome-scale stoichiometric information is mixed with carbon transitions information (used in 2S-^13^C MFA). Examples of these types of networks along with tutorials on how to use these classes to manipulate them can be found in Jupyter notebook A3.

#### FluxModels

The FluxModels module (Fig. 3, and Additional file 1: Figure S2) contains the classes used to solve each of the various types of flux problems solved in this library: FBA (*FBAModel*), ^13^C MFA (*C13Model*), 2S-^13^C MFA (*TwoSC13Model*) and ELVA (External Labeling Variability Analysis, *ELVAModel*, a subproblem of 2S-^13^C MFA, [23]). Flux Variability Analysis (FVA [36]) and ^13^C Flux Variability Analysis (^13^C FVA [23]) can be solved from the same model as for FBA and ^13^C MFA respectively, since they require the same information. Aditionally, classes for the results obtained from each of these types of models are included, which are used to plot, analyze and manipulate results (*FBAResults*, *FVAResults*, *C13Results*, *TSResults*, *ELVAResults*, *C13FVAResults* and *TSFVAResults*). More details can be found in Jupyter notebook A4.

#### EnhancedLists

The enhancedLists module (Additional file 1: Figure S3) holds lists that have been enhanced with methods useful for the objects contained. In this way, there is e.g. a *ReactionList* class that holds reactions and e.g. has a *carbonTransitionsOK()* method that tests if carbon transition and reaction information are compatible (same reactants, same products, etc). The types of lists included are *ReactionList*, *MetaboliteList*, *EMUList*, *EMUTransitionList* and *AtomTransitionList*. Their use is demonstrated in Jupyter notebook A2.

#### GAMSclasses

The GAMSclasses module contains the classes used to solve the optimization problems for each model (see below) through the GAMS modeling system (GAMS Development Corporation), either in series or in parallel (Fig. 4). As depicted in Fig. 1, all flux techniques require solving an optimization problem using a different type of solver (either CPLEX or CONOPT in GAMS), which are accessed through the GAMS framework. At this moment, this interaction is encoded in this class, but we expect to replace it soon by use of the GAMS python API. More information and tests can be found in Jupyter notebook A5.

#### Predictions

This module provides the classes needed to make flux predictions using the Minimization of Metabolic Adjustment (MoMA [36]) and Regulatory on/off minimization (ROOM [37]) methods that were used in the original 2S-^13^C MFA paper [23]. A demonstration can be found in Jupyter notebook A6.

#### Labeling

The Labeling module contains all classes needed to analyze, manipulate and plot labeling patterns (a.k.a Mass Distribution Vector, MDV, the fraction of molecules with *n* =1, 2, 3... labeled carbons) and fragments. The base class *labelingData* is used to derive *LCMSLabelData*, *CEMSLabelData* and *GCMSLabelData*, which are classes for each type of mass spectroscopy data. The *fragment* class contains all information for the MS fragment harboring the MDV and is used to derive classes *GCMSfragment*, *LCMSfragment* and *CEMSfragment*. More details can be found in Jupyter notebook A7.

#### DB

The DB module houses a database of all hard-coded information, conveniently located in a single module. This includes, for example, a dictionary of fragments with all their detailed information: name, abbreviation, formula, number of carbons, formula without carbon backbone, etc.

#### Utilities

The utilities module stores small functions that are useful in a variety of modules: e.g. a function to extract the atoms numbers from an elemental composition string (^′^ *H6NO2Si* ^′^⇒ [ (^′^ *H* ^′^,6),(^′^ *N* ^′^,1),(^′^ *O* ^′^,2),(^′^ *Si* ^′^,1)]).

## Results and discussion

We will now demonstrate the use of the jQMM library for each of the techniques included in the package using data from the literature.

### ^13^*C* Metabolic Flux Analysis for TCA toy model

**Fig. 5:**
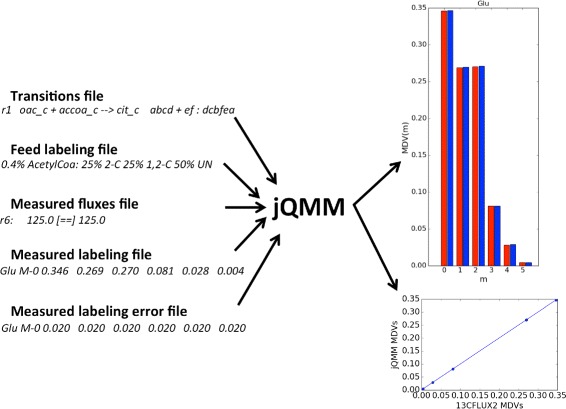
Example jQMM inputs and outputs for TCA toy model. The TCA toy model represents a symplification of the TCA cycle that still retains its main features and is used to test ^13^C MFA tools [35]. In this case, the input consists of the carbon transitions (the fate of each carbon for each the reaction), the feed labeling information (AcetylCoA 50% unlabeled, 25% labeled in the second carbon, 25% labeled in the first two carbons), the measured labeling patterns (glutamate MDVs), the error in these measurements, and the fluxes which are known (e.g. r6). File *line* examples are given in *cursive below*. In this case, all fluxes are assumed to be measured and the goal is to find the corresponding MDV for glutamate, which is given in the *top right part* of the figure (in *red* experimentally measured MDV, in *blue* theoretically calculated MDV). The *lower right part* of the figure provides a comparison with 13CFLUX2 [29], a well known ^13^C MFA package (See Jupyter Notebook B2)

The first demonstration (Fig. 5) involves the toy model of the TCA cycle originally used by Antoniewicz et al. [35] to showcase the Elementary Metabolite Unit (EMU) method (Fig. 12 in [35]). The toy model is a simplification of the tricarboxylic acid (TCA) cycle that still retains its main features. Acetyl coenzyme A (AcCoA) and aspartate are the two substrates, and glutamate and carbon dioxide are the products. The labeling for glutamate is calculated assuming a mixture of 25% [2-^13^C]AcCoA and 25% [1,2-^13^C]AcCoA as the tracer input (with everything else unlabeled), while aspartate is considered unlabeled. Fluxes are fixed to predetermined values, since the goal in this case is to showcase the calculation of labeling patterns from fluxes. Jupyter Notebook B2 demonstrates how, using jQMM, EMU transitions are generated, and solved through GAMS and CONOPT without the need to decompose the network into decoupled EMU reaction networks. The computed labeling pattern is the same as obtained through 13CFLUX2 [29](see Fig. 5, and Jupyter Notebook B2 for more details).

### ^13^*C* Metabolic Flux Analysis for *E. coli* data

The next demonstration of the jQMM library involves a traditional use of ^13^ *C* MFA to calculate fluxes using the data from Toya et al. [38], in order to reproduce supplementary figure S18 from Garcia Martin et al. [23]. Fluxes are calculated for wild type *E. coli* fitting labeling data for 9 different intracellular metabolites. This calculation uses the original small-scale model for central carbon metabolism that lumps several reactions together and displays only an approximate account of fluxes to biomass. For example, as discussed in Garcia Martin et al. [23], the large fluxes draining acetyl-CoA into biomass and to the exterior of the cell as acetate (each a third of the pyruvate dehydrogenase flux, PDH) imposed in the original publication have been overestimated. The typical biomass function used in these minimal ^13^ *C* MFA models with reactions lumped together involves a specific stoichiometry of central carbon intermediates converted to biomass. However, this represents an approximation since some of the metabolites required for biomass growth are not present in the minimal network model and need to be substituted by their requirements in terms of intermediates considered in the minimal network (acetyl-CoA, in this case). These effects require significant effort to be accurately incorporated into a small-scale model, but they are properly handled by the genome-scale model, as demonstrated in the next example. Jupyter Notebook B3 shows how to calculate fluxes through ^13^ *C* MFA for this case.

### 2S- ^13^*C* Metabolic Flux Analysis for *E. coli* data

**Fig. 6:**
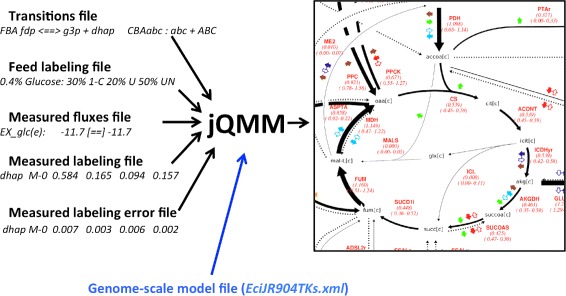
Example jQMM inputs and outputs for E. coli 2S-^13^C MFA. 2S-^13^C MFA allows for the calculation of fluxes for genome-scale models constrained by ^13^C labeling data [23]. The input is the same as for ^13^C MFA(Fig.5), but with the addition of a genome-scale model. The output consists of fluxes for the genome-scale model, which are visualized in a *metabolic map* (see Jupyter Notebook B4)

This example calculates fluxes for a full comprehensive genome-scale model [23] constrained by ^13^C labeling data through the 2S-^13^C MFA algorithm (Fig. 6). The input is the same as for the ^13^C MFA case in the previous section, with the addition of the iJR904 genome-scale model [39]. As discussed in depth in Garcia Martin et al. [23], the solution displays nearly the same values as ^13^C MFA for central metabolism (see e.g. Figure S4 and S18 in [23]). This similarity is expected since 2S-^13^C MFA is designed to mimic ^13^C MFA for this part of metabolism (see “Limiting flux to core reactions” section in [23]). The only difference for the Toya et al. data set can be found in the TCA cycle flux. These differences appear because genome-scale models account for fluxes to biomass in a more realistic manner and because they do not rule out unexpected metabolic routes compatible with the available data.

Furthermore, the use of genome-scale models allows for some information that can not be obtained from smaller, non-comprehensive models, such as an account of all reactions producing and consuming e.g. NADPH as constrained by ^13^C labeling data (Fig. 6 in [23]). Other advantages of using genome-scale models include the prediction of non measured fluxes (Fig. S13 in [23]) and quantitative full predictions of directly measurable data (Fig. 10 in [23]). Jupyter Notebook B4 shows how to reproduce all figures from Garcia Martin et al. [23].

### ^13^*C* Metabolic Flux Analysis for a soil microbial community

In order to show the general capabilities of the jQMM library for non-standard ^13^C MFA we will show how one can use this library to calculate fluxes for microbial communities as shown in Hagerty et al. [25]. The problem presented and solved in this paper is related to ^13^C MFA but, at the same time, the technical details differ notably. In this and other experiments [40, 41], Dijkstra et al. collected several soil samples and incubated them with two different carbon sources (glucose and pyruvate), where each source was chosen to have a different initial labeling (1-^13^C or fully labeled for glucose and 1-^13^C or 2,3-^13^C for pyruvate). For each carbon source and labeling type, the labeling fraction of CO_2_ emmited from the soil was measured. For each carbon source, the two relative CO_2_ labeling profiles provided enough information to fit a small model of carbon metabolism that represented the combination of reactions for all microbial entities in the soil. The good fits supported the assumption that all members of the community display a similar metabolic flux state, and the results are used to derive the carbon utilization efficiency (i.e. how much carbon is diverted into biomass and how much is lost as CO_2_, a critical parameter for climate models [42]). Jupyter notebook B6 shows how to do these calculations using modular combinations of the jQMM library without recourse to complicated excel data sheets [25].

### Flux balance analysis

The jQMM library provides the means to perform FBA (Jupyter notebook B1) and other COBRA methods for the sake of comparison with ^13^C-based methods [23]. However, the main purpose of jQMM is not to provide a COBRA library, since actively developed open source python libraries such as COBRApy [26] and cameo (http://cameo.bio/) are available for this purpose. Methods for FBA, FVA, Minimization of Metabolic Adjustment (MoMA [36]) and Regulatory on/off minimization (ROOM [37]) are provided. In order to showcase these capabilities we replicated part of the tutorial from the COBRA v 1.0 package in Becker et al. [43], which can be found in Jupyter notebook B1. Future versions of the library will likely be changed to use cameo for this functionality.

### Principal Component Analysis of Proteomics (PCAP)

**Fig. 7:**
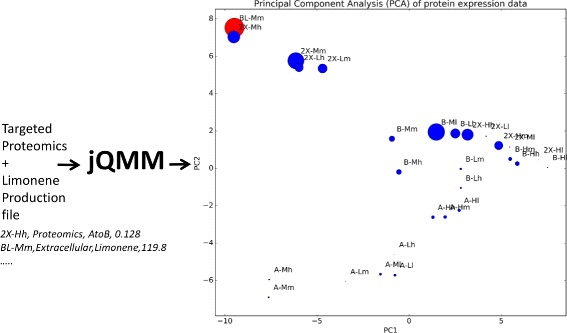
Example jQMM inputs and outputs for Principal Component Analysis of Proteomics (PCAP). PCAP is a method developed to leverage proteomics data in order to improve bioproduct yield. The inputs are measured proteomic profiles and bioproduct production levels and the output consists of a *plot* that can be used to increase yields as shown in Alonso-Gutierrez et al. [24]. (see Jupyter Notebook B5). File *line* examples are provided in *cursive below*

While most of the jQMM library is focused on flux analysis, we have found it useful to statistically model -omics data without the recourse to mechanistic models [24], an approach which we expect to be increasingly popular in the coming years given the increasing availability of -omics data. In Jupyter notebook B5, we show how to use the jQMM library and quantitative proteomics data to increase biofuel yields by replicating the PCAP (Principal Component Analysis for Proteomics) analysis with a recreation of previously published figures [24] (Fig. 7).

This case represents a prototypical example of pathway engineering, in which a series of exogenous genes from a variety of organisms (*E. coli*, *S. cerevisiae*, *S. aureus*, and plants such as *M. spicata*) were assembled together in a pathway able to produce limonene [44], a jet-fuel [45]. However, when assembling this pathway together, there was no easy way to figure out the design parameters for optimal function: what promoter strength to use, when to induce? how much? In this case, a combinatorial approach was used, using promoters of different strength (weak, medium, strong), different induction strengths (low, medium, high) and induction times (low, medium, high). For each of these scenarios, limonene production and protein expression for all genes in the pathway were measured. The raw proteomics data did not provide clear trends but the use of a basic data analysis tool such as a Principal Component Analysis (PCA) produced actionable results which allowed for the improvement of limonene production by 40% and bisabolene production by 200% leading to the highest producing strain of this biodiesel replacement to that date [24]. Jupyter notebook B5 shows how to perform this analysis.

## Conclusion

We have presented here the jQMM library, an open-source modular library for modeling metabolism and the first open-source library for ^13^C MFA in Python. The jQMM library is also the first one to provide a method to calculate fluxes for genome-scale models constrained by ^13^C labeling data through 2S-^13^C MFA, and is the first to unify the FBA, ^13^C MFA and 2S-^13^C MFA in a single package. Unlike METRAN [27], INCA [28] and 13CFLUX2 [29]), it is open source and, unlike OPENFLUX [30] and OPENFLUX2 [31], it is written in Python. Furthermore, it includes an example on how to calculate fluxes for microbial communities through ^13^C MFA, and the tools to leverage other types of -omics data (e.g. proteomics for biofuel production).

While the examples here are mainly focused on various types of flux analysis (FBA, ^13^C MFA, 2S-^13^C MFA, etc) and biofuels, the tools are of general aplicability to other fields (e.g. pharma, biomedicine, environmental microbiology) and include statistical methods as well (e.g. PCAP). jQMM presents not only methods for modeling and generating actionable items form -omics data, but also libraries for data manipulation and visualization (fluxes and labeling patterns see Figs. 6 and 5), including classes for most common concepts in metabolism (e.g. reactions, metabolites, GC-MS fragments, reaction networks) in order to facilitate their manipulation, and which are of utility beyond the examples presented here. Every module in jQMM is explained in detail in Jupyter notebooks that allow for immediate reproducibility. Python unit tests are included for all modules for ease of testing when adding functionality.

jQMM will continue growing as the methods developed and used at JBEI for biofuel production expand and mature, while other authors contribute to it. At the moment jQMM is heavily dependent on the closed-source commercial platform GAMS and the CPLEX and CONOPT solvers. Our first future improvement to the jQMM library is planned to involve providing an interface to open-source and free version of solvers equivalent to GAMS. Further future improvements will likely include integration with COBRApy [26], cameo (cameo.bio) or KBase (kbase.us).

## Availability of data and materials

**Project name:** jQMM library

**Project home page:** https://github.com/JBEI/jqmm

**Operating systems:** Platform independent

**Programming language:** Python, GAMS

**Other requirements:** CONOPT large scale nonlinear solver

All data used in this paper can be found in the github library itself. Additional file 1 contains a mathematical description of the algorithms, and Figures S1-S3.

## Additional file

Additional file 1 Supplementary file containing the mathematical description of algorithms, and supplementary figures S1-S3. (PDF 2250 kb)

## Abbreviations

^13^C MFA: ^13^C Metabolic Flux Analysis
2S-^13^C MFA: Two-scale ^13^C Metabolic Flux Analysis
AcCoa: Acetyl-CoA
API: Application Program Interface
COBRA: COnstraint Based Reconstruction and Analysis
ELVA: External Labeling Variability Analysis
EMU: Elementary Metabolite Unit
FBA: Flux Balance Analysis
FVA: Flux Variability Analysis
JBEI: Joint BioEnergy Institute
jQMM: JBEI Quantitative Metabolic Modeling
MDV: Mass Distribution Vector
MoMA: Minimum of Metabolic Adjustment
NADPH: Nicotinamide adenine dinucleotide phosphate
PDH: Pyruvate Dehydrogenase
PCAP: Principal Component Analysis of Proteomics
ROOM: Regulatory On/Off Minimization
TCA: trycarboxylic acid
