# Erratum to: The JBEI quantitative metabolic modeling library (jQMM): a python library for modeling microbial metabolism

**DOI:** 10.1186/s12859-017-1631-y

**Published:** 2017-04-18

**Authors:** Garrett W. Birkel, Amit Ghosh, Vinay S. Kumar, Daniel Weaver, David Ando, Tyler W. H. Backman, Adam P. Arkin, Jay D. Keasling, Héctor García Martín

**Affiliations:** 1 Biological Systems and Engineering Division, Lawrence Berkeley National Laboratory, Berkeley, CA USA; 2 Joint BioEnergy Institute, Emeryville, CA USA; 3 Department of Chemical and Biomolecular Engineering, University of California, Berkeley, CA USA; 4 Department of Bioengineering, University of California, Berkeley, CA USA; 5 Environmental Genomics and Systems Biology Division, Lawrence Berkeley National Laboratory, Berkeley, CA USA; 6 School of Energy Science and Engineering, Indian Institute of Technology (IIT), Kharagpur, India; 7 Novo Nordisk Foundation Center for Biosustainability, Technical University of Denmark, DK2970 Hørsholm, Denmark; 8 DOE Agile BioFoundry, Emeryville, CA USA; 9 BCAM, Basque Center for Applied Mathematics, Bilbao, Spain

## Erratum

Following publication of this article [1], it has come to our attention that an incomplete version of Fig. 7 was included in this article. The complete figure is shown below with the missing text included to the left of the chart.

**Fig. 7:**
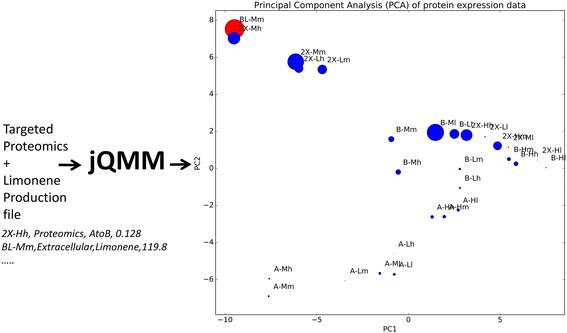
Principal component analysis (PCA) of protein expression data
