# Supplementary material for: The JBEI quantitative metabolic modeling library (jQMM): a python library for modeling microbial metabolism
Source: BMC Bioinformatics. 2017 Apr 5;18:205. doi: 10.1186/s12859-017-1615-y (PMC5382524; doi:10.1186/s12859-017-1615-y)
Supplement: Additional file 1 — Supplementary file containing the mathematical description of algorithms, and supplementary figures S1-S3. (PDF 2250 kb) [file 12859_2017_1615_MOESM1_ESM.pdf]

SOFTWARE

The JBEI Quantitative Metabolic Modeling library (jQMM): a Python library for modeling microbial metabolism. SUPLEMENTARY MATERIAL

Garrett W Birkel[1,2,8], Amit Ghosh[1,2,6], Vinay S Kumar[1,2], Daniel Weaver[1,2], David Ando[1,2], Tyler W H Backman[1,2,8], Adam P Arkin[1,4,5], Jay D Keasling[1,2,3,4,7] and Héctor García Martín[1,2,8,9]*

*Correspondence: hgmartin@lbl.gov
[1]Biological Systems and Engineering Division, Lawrence Berkeley National Laboratory, Berkeley CA, USA
Full list of author information is available at the end of the article

Mathematical description of algorithms

This section describes the inputs, outputs and optimization problems for each of the flux analysis techniques used: FBA, ^{13}C MFA and 2S-^{13}C MFA. Mathematical details for PCAP analysis of proteomic data can be found in Jupyter notebook B5 and in the original publication[1].

Flux Balance Analysis

The mathematical details for FBA are the same as for other implementations[2], and are provided here for comparison with other methods. We only implemented them in jQMM so as to provide an equivalent comparison in situations like Fig. 9 in Garcia Martin *et al*[3].

Input

The inputs for FBA are: a genome-scale model, measurements for exchange fluxes (e.g. glucose input, acetate output, etc), and an objective function (typically growth rate maximization).

Optimization problem

$$\text{Maximize} \quad v_{obj} \tag{1}$$

Subject to:

$$\sum_j S_{ij} v_j = 0 \qquad \forall i \in I^N, j \in J \tag{2}$$

$$lb_j \leq v_j \leq ub_j \qquad \forall j \in J \tag{3}$$

where:

Sets

$I^N \subset I$	: Set of non-exchange metabolites.
$J = \{j\}$	: Set of fluxes.

Parameters

S_{ij}	: Stoichiometry matrix.
ub_i, lb_i	: Upper and lower bounds for reaction i.

Variables

v_i	: Flux value of reaction i, in mmol/gdw/h.

and obj is the objective flux, for maximum growth rate $obj = BiomassEcoli$ and for ATP maximization $obj = ATPM$[4].

Output

The output involves the flux profile v_j $\forall j \in J$

^{13}C Metabolic Flux Analysis

^{13}C MFA equations are included here because they are slightly different to the equations used in Garcia Martin *et al*[3]. The results are the same, but the new equations are able to handle reactions with repeated reactants.

Input

The inputs for ^{13}C MFA are a set of transitions (e.g. `PDH: pyr --> co2 + accoa ; abc --> a + bc` as explained above) and measurements for exchange fluxes (as for FBA above).

Optimization problem

$$\text{Minimize } OF = \sqrt{\sum_{e \in E_{\text{meas}}} \left(\sum_{m \in M_e} \left(\frac{f_{em}^{exp} - f_{em}}{\Delta_{em}} \right)^2 \Big/ |M_e| \right) \Big/ |E_{\text{meas}}|} \quad (4)$$

Subject to:

$$\sum_j S^*_{ij} V_j = 0 \qquad \forall i \in I^N, j \in J^B \quad (5)$$

$$LB_j \leq V_j \leq UB_j \qquad \forall j \in J^B \quad (6)$$

$$\sum_{m \in M_e} f_{em} = 1 \qquad \forall e \in E \quad (7)$$

$$\sum_{e' \in E} \left(\left(\sum_{j | EMM^j_{e'->e} > 0} EMM^j_{e'->e} S^*_{ij} V_j \right) f_{e'm} \right) + \left(\sum_{j | S^*_{ij} < 0} S^*_{ij} V_j \right) f_{em} = 0 \qquad \forall m \in M_e, e \in E_i, i \in I^N \quad (8)$$

$$f_{em} = \sum_{w \in W_{em}} \prod_{n=1}^{|E_e|} f_{e_n m_n} \qquad \forall m \in M_e, e \in E^c \quad (9)$$

where:

Sets

$I \equiv \{i\}$	: Set of all metabolites.
$I^N \subset I$	: Set of non-exchange metabolites.
J^B	: Set of fluxes with backward and forward fluxes differentiated, e.g. PGI_f, PGI_b, PGL .... etc.
$E = \{e\}$	: Elementary Metabolite Units (EMUs).
$E^c \subset E$	: Combined EMUs.
$E_i \subset E$	: EMUs from metabolite $i \in I$.
$E_e \subset E$	: EMUs that produce combined EMU e.
$E_{meas} \subset E$	: EMUs corresponding to measured EMUs.
W_{em}	: Set of every possible mass isotopomer multiplet of E_e that produce the mass isotopomer m of e.
M_e	: m values for MDV of emu $e : 0, 1, \ldots, \#$ of carbons in e .

Parameters

$EMM^j_{e'->e}$	$: = \frac{1}{k}$ if e' produces e through reaction $j \in J^B$, 0 otherwise. See below.
S^*_{il}	: Stoichiometry matrix with backward and forward fluxes differentiated.
UB_j, LB_j	: Upper and lower bounds for reaction j.
$f^{exp}_{em} \in [0, 1]$	: Experimentally measured MDV(m)[5] for emu e.
Δ_{em}	: Measurement error for f^{exp}_{em}.

Variables

V_l	: Flux value of reaction $i \in J^B$, normalized to glucose input rate.
$f_{em} \in [0, 1]$	: MDV for emu e from metabolite $m \in M_e$.

Output

The output involves a flux profile v_j $\forall j \in J$ with confidence intervals. For example: `PDH: [ 0.3 :0.4 : 0.6 ]` Where 0.5 mmol/gdw/hr is the best fit for the data, and 0.3 mmol/gdw/hr and 0.6 mmol/gdw/hr are the minimum and maximum flux values compatible with the labeling data as determined through ^{13}C FVA[3].

2S-^{13}C Metabolic Flux Analysis

2S-^{13}C MFA is a hybrid of FBA and ^{13}C MFA where the stoichiometry constraints are applied to the full genome-scale network, as in the case of FBA, and the labeling constraints are applied only to the core set of reactions and metabolites, as is the case for ^{13}C MFA[3].

Input

The inputs for 2S-^{13}C MFA are: a genome-scale model, a set of transitions and measurements for exchange fluxes.

Optimization problem

In the notation of [6]:

$$\text{Minimize } OF = \sqrt{\left(\sum_{\substack{e \in E_{meas} \\ m \in M_e}} \left(\frac{f_{em}^{exp} - f_{em}}{\Delta_{em}} \right)^2 / |M_e| \right) / |E_{meas}|} \tag{10}$$

Subject to:

$$\sum_j S_{ij} v_j = 0 \qquad \forall i \in I^N, j \in J \tag{11}$$

$$lb_j \leq v_j \leq ub_j \qquad \forall j \in J \tag{12}$$

$$\sum_{m \in M_e} f_{em} = 1 \qquad \forall e \in E_{co} \tag{13}$$

$$\sum_{e' \in E_{co}} \left(\left(\sum_{l | EMM^l_{e'->e} > 0} EMM^l_{e'->e} S^*_{il} V_l \right) f_{e'm} \right) + \left(\sum_{l | S^*_{il} < 0} S^*_{il} V_l \right) f_{em} = 0 \qquad \forall m \in M_e, e \in E_i, i \in I^N_{co} \tag{14}$$

$$f_{em} = \sum_{w \in W_{em}} \prod_{n=1}^{|E_e|} f_{e_n m_n} \qquad \forall m \in M_e, e \in E^c_{co} \tag{15}$$

$$v_j = \sum_{l \in J^B_{co}} map_{jl} V_l \qquad \forall j \in J \tag{16}$$

where:

<u>*Sets*</u>

$I \equiv \{i\}$	: Set of all metabolites.
$I_{co} \subset I$	: Set of core metabolites.
$I_{co}^{N} \subset I_{co}$	: Set of non-exchange core metabolites.
J	: Set of fluxes.
$J_{co} \subset J$	: Set of core fluxes.
J^{B}	: Set of fluxes with backward and forward fluxes differentiated, e.g. PGI_f, PGI_b, PGL .... etc.
$J_{co}^{B} \subset J^{B}$	: Set of core fluxes for J^{B} .
$E = \{e\}$	: Elementary Metabolite Units (EMUs).
$E^{c} \subset E$	: Combined EMUs.
$E_{i} \subset E$	: EMUs from metabolite $i \in I$.
$E_{co}^{c} \subset E^{c}$	: Core combined EMUs.
$E_{e} \subset E$	: EMUs that produce combined EMU e.
$E_{co} \subset E$	: EMUs corresponding to core metabolites.
$E_{meas} \subset E$	: EMUs corresponding to measured EMUs.
W_{em}	: Set of every possible mass isotopomer multiplet of E_{e} that produce the mass isotopomer m of e.
M_{e}	: m values for MDV of emu e : $0, 1, \ldots, \#$ of carbons in e .

<u>*Parameters*</u>

$EMM_{e'->e}^{l}$	$= \frac{1}{k}$ if e' produces e through reaction $l \in J_{co}^{B}$, 0 otherwise. See [6] .
S_{ij}	: Stoichiometry matrix.
S_{il}^{*}	: Stoichiometry matrix with backward and forward fluxes differentiated.
ub_{j}, lb_{j}	: Upper and lower bounds for reaction j.
$f_{em}^{exp} \in [0,1]$	: Experimentally measured MDV for emu e from metabolite m.
Δ_{em}	: Measurement error for f_{em}^{exp}.
map_{jl}	$= 1 * glucupt$ if l corresponds to forward flux of j.
	$= -1 * glucupt$ if l corresponds to backward flux of j.

<u>*Variables*</u>

v_{j}	: Flux value of reaction $j \in J$, in mmol/gdw/h.
V_{l}	: Flux value of reaction $l \in J_{co}^{B}$, normalized to glucose input rate.
$f_{em} \in [0,1]$	: Mass isotopomer fraction (MDV) for emu e from metabolite m.

Notice that S_{ij}^{*} is not the same as S_{ij}, since J and J^{B} are slightly different sets of fluxes. In fact:

$$
\begin{aligned}
S_{il}^{*} &= S_{ij} \quad \text{if } l \text{ is the forward version of } j. \\
S_{il}^{*} &= -S_{ij} \quad \text{if } l \text{ is the backward version of } j.
\end{aligned}
\tag{17}
$$

Output

The output involves a flux profile $v_{j}\ \forall j \in J$ for all reactions in the genome-scale model with confidence intervals.

Author details

[1]Biological Systems and Engineering Division, Lawrence Berkeley National Laboratory, Berkeley CA, USA. [2]Joint BioEnergy Institute, Emeryville CA, USA. [3]Department of Chemical and Biomolecular Engineering, University of

California, Berkeley, CA, USA. [4]Department of Bioengineering, University of California, Berkeley, CA, USA. [5]Environmental Genomics and Systems Biology Division, Lawrence Berkeley National Laboratory, Berkeley CA, USA. [6]School of Energy Science and Engineering, Indian Institute of Technology (IIT), Kharagpur, India. [7]Novo Nordisk Foundation Center for Biosustainability, Technical University of Denmark, DK2970-Hørsholm, Denmark. [8]DOE Agile BioFoundry, Emeryville CA, USA. [9]BCAM, Basque Center for Applied Mathematics, Bilbao, Spain.

References

1. Alonso-Gutierrez, J., Kim, E.-M., Batth, T.S., Cho, N., Hu, Q., Chan, L.J.G., Petzold, C.J., Hillson, N.J., Adams, P.D., Keasling, J.D., Garcia-Martin, H., Soon Lee, T.: Principal component analysis of proteomics (PCAP) as a tool to direct metabolic engineering. Metabolic Engineering **28**, 123–133 (2014). doi:10.1016/j.ymben.2014.11.011
2. Ebrahim, A., Lerman, J.A., Palsson, B.O., Hyduke, D.R.: COBRApy: COnstraints-Based Reconstruction and Analysis for Python. BMC Systems Biology **7**(1), 74 (2013). doi:10.1186/1752-0509-7-74
3. Garcia Martin, H., Kumar, V.S., Weaver, D., Ghosh, A., Chubukov, V., Mukhopadhyay, A., Arkin, A., Keasling, J.D.: A Method to Constrain Genome-Scale Models with 13C Labeling Data. PLoS Computational Biology **11**(9) (2015). doi:10.1371/journal.pcbi.1004363
4. Ramakrishna, R., Edwards, J.S., McCulloch, A., Palsson, B.O.: Flux-balance analysis of mitochondrial energy metabolism: consequences of systemic stoichiometric constraints. American journal of physiology. Regulatory, integrative and comparative physiology **280**(3), 695–704 (2001)
5. Suthers, P.F., Burgard, A.P., Dasika, M.S., Nowroozi, F., Van Dien, S., Keasling, J.D., Maranas, C.D.: Metabolic flux elucidation for large-scale models using 13C labeled isotopes. Metabolic Engineering **9**(5-6), 387–405 (2007). doi:10.1016/j.ymben.2007.05.005. NIHMS150003
6. Suthers, P.F., Chang, Y.J., Maranas, C.D.: Improved computational performance of MFA using elementary metabolite units and flux coupling. Metabolic Engineering **12**(2), 123–128 (2010). doi:10.1016/j.ymben.2009.10.002

Supplementary Figures

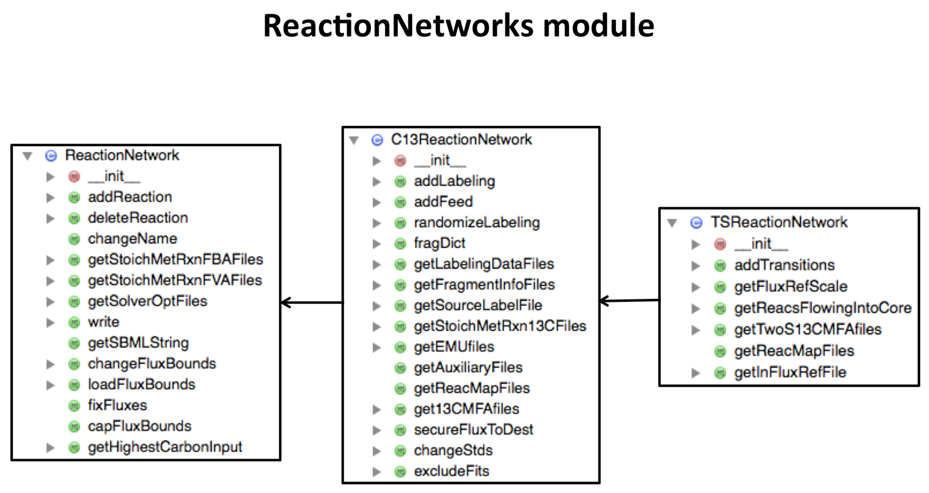

Figure S1: **ReactionNetworks module class diagram.** The ReactionNetworks modules contains classes for the three types of networks in the jQMM library: stoichiometric networks (*reactionNetwork*, used for FBA), stoichiometric networks with carbon transitions (*C13reactionNetwork*, for ^{13}C MFA) and stochiometric with carbon transitions only for a defined core (*TSreactionNetwork*, for 2S-^{13}C MFA).

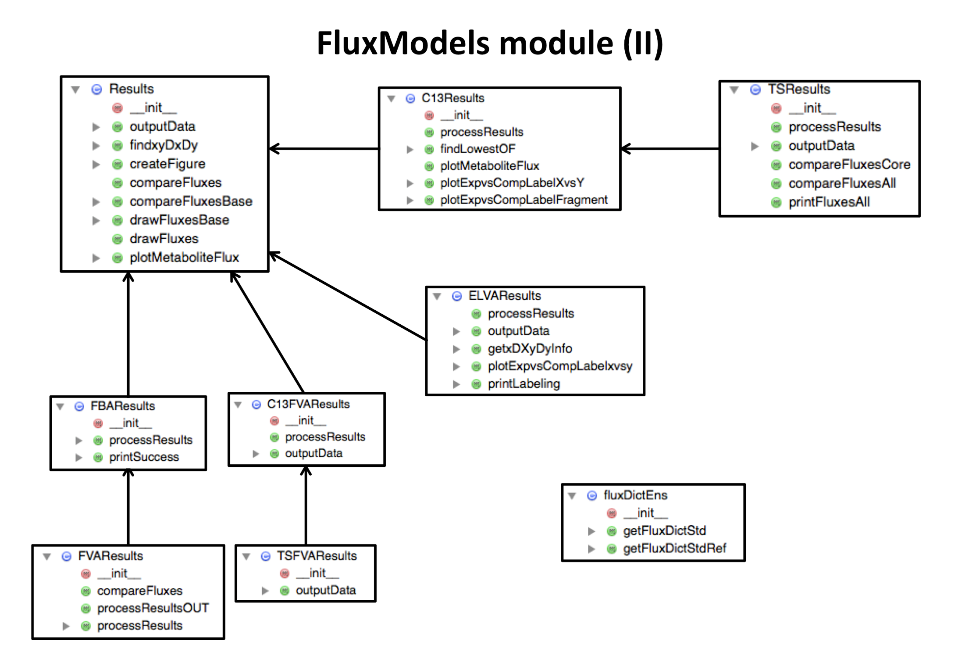

Figure S2: **FluxModels module class diagram (part II).** The FluxModels module also contains classes for storing results for each of the flux analysis types. Arrows indicate derived classes.

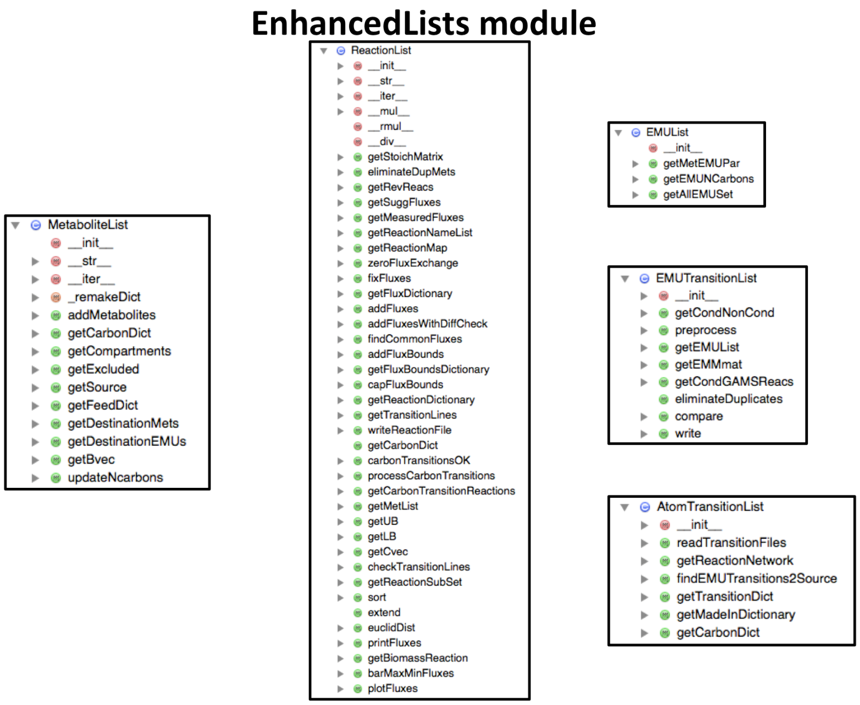

Figure S3: **EnhancedLists module class diagram.** The EnhancedLists module involves lists of special objects from core. For example, a reactionList class is provided that is a list of reactions enhanced with a variety of useful functions. Other enhanced lists include list of metabolites, emus, atom transitions and emu transitions.
